# Surface Enhanced Raman Scattering on Regular Arrays of Gold Nanostructures: Impact of Long-Range Interactions and the Surrounding Medium

**DOI:** 10.3390/nano10112201

**Published:** 2020-11-04

**Authors:** Iman Ragheb, Macilia Braïk, Stéphanie Lau-Truong, Abderrahmane Belkhir, Anna Rumyantseva, Sergei Kostcheev, Pierre-Michel Adam, Alexandre Chevillot-Biraud, Georges Lévi, Jean Aubard, Leïla Boubekeur-Lecaque, Nordin Félidj

**Affiliations:** 1 Université de Paris, Laboratoire ITODYS, CNRS, F-75006 Paris, France; imanraghebomar@gmail.com (I.R.); stephanie.lau@univ-paris-diderot.fr (S.L.-T.); alexandre.chevillot@univ-paris-diderot.fr (A.C.-B.); georges.levi@univ-paris-diderot.fr (G.L.); jean.aubard@univ-paris-diderot.fr (J.A.); leila.boubekeur@univ-paris-diderot.fr (L.B.-L.); 2 Université Mouloud Mammeri de Tizi-Ouzou, LPCQ, BP 17 RP, 15000 Tizi-Ouzou, Algeria; massiliabraik@gmail.com (M.B.); belabd2000@yahoo.fr (A.B.); 3 Charles Delaunay Institute, P2MN Department, University of Technology of Troyes, CS 42060, 10004 Troyes, France; anna.rumyantseva@utt.fr (A.R.); sergei.kostcheev@utt.fr (S.K.); pierre_michel.adam@utt.fr (P.-M.A.)

**Keywords:** localized surface plasmon, surface enhanced Raman scattering, grating effect, gold nanodisks, Rayleigh anomaly

## Abstract

Long-range interaction in regular metallic nanostructure arrays can provide the possibility to manipulate their optical properties, governed by the excitation of localized surface plasmon (LSP) resonances. When assembling the nanoparticles in an array, interactions between nanoparticles can result in a strong electromagnetic coupling for specific grating constants. Such a grating effect leads to narrow LSP peaks due to the emergence of new radiative orders in the plane of the substrate, and thus, an important improvement of the intensity of the local electric field. In this work, we report on the optical study of LSP modes supported by square arrays of gold nanodiscs deposited on an indium tin oxyde (ITO) coated glass substrate, and its impact on the surface enhanced Raman scattering (SERS) of a molecular adsorbate, the mercapto benzoic acid (4-MBA). We estimated the Raman gain of these molecules, by varying the grating constant and the refractive index of the surrounding medium of the superstrate, from an asymmetric medium (air) to a symmetric one (oil). We show that the Raman gain can be improved with one order of magnitude in a symmetric medium compared to SERS experiments in air, by considering the appropriate grating constant. Our experimental results are supported by FDTD calculations, and confirm the importance of the grating effect in the design of SERS substrates.

## 1. Introduction

Over the two last decades, metallic nanostructures led to a lot of research in nano-optics, thanks to their unique plasmonic properties [1]. These properties are connected to localized surface plasmon (LSP) resonances associated to collective oscillations of conductive electrons at the surface of the nanoparticles (NPs) [2]. The LSP wavelength depends on the geometrical parameters of the NPs, the chemical composition of the metallic NPs, the inter-particles distance and the surrounding medium [3,4]. In addition, these optical proprieties are characterized by a strong extinction in the far-field in the visible and near-infrared range (mainly for gold and silver), and a strong electric field enhancement in the near-field of the nanostructures [5].

Depending on the distance between nanoparticles, two coupling modes can be considered: a short-range coupling in the near-field of the particles and a long-range coupling [6,7,8,9]. The short-range coupling occurs when the separation distance *d* is much smaller than the optical wavelength *λ* (typically *d* smaller than 10 nm). The particles are thus treated as dipoles interacting through their near-field [10]. Near-field coupling results from the Coulomb interaction between the surface charges on particles and becomes stronger when the areas presenting a high charge density are close to each other, and increases when the distance between nanoparticles is reduced. This type of coupling exhibits large charge dipoles particularly in the gap between nanoparticles, leading to strong local fields compared to the case of isolated nanoparticles [11]. As a result, the separation distance strongly affects the optical response of the system. For instance, when distance between two nanodiscs decreases, the LSP resonance is red-shifted due to the decreasing of the restoring force for single nanoparticles (for a polarization parallel to the main axe of the NP dimer) [12]. As a consequence, the splitting energy between the new hybridized modes is increased and the coupling becomes stronger. In addition, this coupling has a strong impact on the near-field of the nanoparticles. Compared to a single nanoparticle, a dimer of nanoparticles exhibits a higher electric field enhancement due to the dipoles interaction between the plasmon modes, mainly located in the gap between the nanoparticles (called hot-spots) [13,14].

A long-range interaction in regular metallic nanostructure arrays can also provide the possibility to manipulate their optical properties [15,16]. When assembling the nanoparticles in an array, interactions between nanoparticles can result in long-range interactions, for specific inter-particle distances (grating constant) [17,18]. As a result, the optical response exhibits a narrow LSP peak due to the emergence of radiative orders in the plane of the substrate [19,20,21,22]. Plasmonic nanostructures arranged in regular arrays support lattice (or collective) plasmon modes, and the interference between localized surface plasmon (LSP) and the so-called Rayleigh anomaly leads to a reduced linewidth of the resonance, and thus, an important improvement of its quality factor [23,24]. Such effect finds applications in non-linear optics [25], molecular sensing [26], plasmon-based lasers [27], surface enhanced fluorescence [28] and surface enhanced Raman scattering (SERS) [29,30,31].

Only a very few works related to long-range interactions have been dedicated to this field. The SERS effect originates mainly from an electromagnetic enhancement mechanism consecutive to the excitation of localized surface plasmon (LSP) of metallic nanoparticles, and takes place for molecules (including at very low concentration) close to the surface of metallic particles, provided that the laser line wavelength is close to the maximum of LSP resonance [32,33]. In particular, molecules adsorbed in the first surface layer display the largest Raman enhancement factors (REF). Taking into account both enhanced fields, the average Raman gain <G> can be expressed as [34]:

(1) $$<G>=\langle {\left|A(\nu_{exc})\right|}^{2}\times {\left|A(\nu_{R})\right|}^{2}\rangle$$

where $A(\nu_{exc})$ is the local electric-field enhancement factor at the incident frequency $\nu_{exc}$, and $A(\nu_{R})$ is the corresponding factor at the Raman frequency $\nu_{R}$. Most of the time, $\langle G\rangle$ is averaged over the surface area of the particles, in order to estimate the Raman gain. In general, $\langle G\rangle$ is approximated by assuming that $A(\nu_{exc})$ and $A(\nu_{R})$ are identical; hence, $\langle G\rangle$ can be rewritten $\langle G\rangle ∼{\left|A(\nu_{exc})\right|}^{4}$ [35]. This approximation takes advantage of the fact that the LSP width is often large compared to the Stokes shift, except for calibrated samples like lithographic structures, where the LSP band can be narrow [36].

Recently, directional plasmon excitation and SERS studies have been investigated for arrays of gold lines deposited on a gold film [37]. The excitation of the surface plasmon polariton (SPP) takes place either at the metal-air interface or the metal-glass interface leading to the appearance of diffractive modes. Such configuration, although interesting, prevented to estimate easily Raman gains, due to the roughness of the gold film, contributing also the Raman enhancement of the molecular probes. In this work, we report on the optical study of LSP modes supported by square arrays of gold nanodiscs deposited on a indium tin oxyde (ITO) coated glass substrate, and its impact on the SERS response of a molecular adsorbate, the mercapto benzoic acid (4-MBA). We estimated the Raman gain of these molecules, by varying the grating constant and the refractive index of the surrounding medium of the superstrate, from an asymmetric medium (air) to a symmetric medium (oil) with respect to the substrate. We show that the Raman gain can be improved with one order of magnitude in a symmetric medium compared to SERS experiments in air, by considering the appropriate grating constant in accordance with FDTD calculations. They confirm the importance and the impact of the grating effect in the design of SERS substrates.

## 2. Results and Discussion

**Figure 1:**
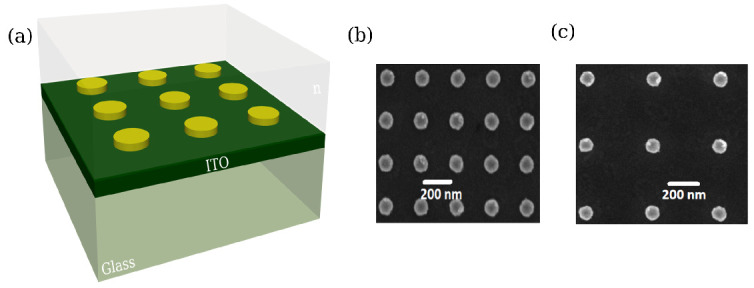
(**a**) Scheme of the gold nanoparticle (NP) array deposited on an indium tin oxyde (ITO) (thickness 80 nm) coated glass substrate and surrounded with a dielectric of refractive index n. (**b**,**c**) SEM images of gold nanodisc square arrays with a diameter of 100 nm and a grating constant Λ of 250 nm and 450 nm, respectively. Height of the discs h = 50 nm.

Gold nanodiscs arrays (size of 60 × 60 μm^2^) were fabricated by electron beam lithography (EBL). The gold nanodiscs height and diameter have been fixed to h = 50 nm and D = 100 nm, respectively. The grating constant (inter-particle distance center-to-center) is varying from Λ = 250 nm to 550 nm. As seen in the Figure 1, the arrays are homogeneous in term of grating constant. Several configurations can be considered depending on the index of the over layer (upper medium).

- The upper medium is air with n = 1 index. This configuration leads to an asymmetric environment since the index of the ITO substrate varies from n = 1.9 to n = 1.7 in the wavelength range.
- The upper medium is water with index n ≃ 1.33 enabling a partial matching with the ITO substrate index.
- The upper medium oil matching index of n ≃ 1.55 leading to a better matching with ITO index.

It has been shown theoretically and experimentally that matched indices improves greatly the grating effect [8,18].

**Figure 2:**
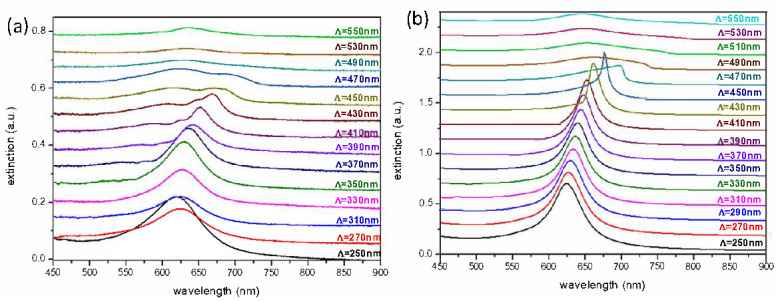
(**a**) Experimental and (**b**) calculated extinction spectra in air (in transmission, light at normal incidence and polarized along the x-axis) for square arrays of gold nanodiscs with a diameter D = 100 nm. The grating constant is varying from Λ = 250 to 550 nm with steps of 20 nm. The height of nanodiscs is fixed to h = 50 nm. The calculations have been obtained by the FDTD method.

We first focus our attention on the extinction spectra and SERS experiments on square arrays of gold discs in air, with grating constants varying from Λ = 250 to 550 nm (see Figure 2). For short grating constants, the LSP position is very close to the LSP resonance of isolated nanoparticles. Indeed, no diffracted order is observed in such situation. Figure 2b displays the calculated extinction spectra for a grating constant using the FDTD method. The experimental spectra are in very good qualitative agreement with the calculated ones, although with a smaller full width at half maximum (FWHM) and slightly blue-shifted for the calculated ones, due to the fact that, in the calculations, the nanoscale surface roughness (NSR) of the gold discs was not taken into account [38,39]. When the grating constant is increased, a significant red-shift of the LSP is expected, as well as a reduced FWHM. This optical behavior is confirmed by the calculated extinction spectra when varying the grating constant, as displayed in the Figure 2b. The extinction spectra are attributed to collective LSP resonances (so-called lattice modes).

According to the grating theory, for a grating constant of Λ_*c*_ with a refractive index of the substrate n_*sub*_, the position of the Rayleigh anomaly is given by Λ_*c*_ = *λ*/n_*sub*_, for an illumination at normal incidence. In the range of LSP wavelengths and grating constants considered in Figure 2, the Rayleigh anomaly can be excited, corresponding to (±1,0) diffraction order in the plane of the substrate. Therefore, when the lattice wavelength is close to the positions of the Rayleigh anomaly, a reduced FWHM is observed. This behavior is due to a strong coupling between the LSP mode and the Rayleigh anomaly, which is observed for a critical grating constant Λ_*c*_ = 410 nm in our experiments (Figure 2a). For such grating constant (Λ = 410 nm), a reduced FWHM with a quality factor of Q_*c*_ = 18.75 is measured, and higher compared to the case of Λ = 270 nm, for which Q = 8.68 (the quality factor is defined as Q = *ω*/Δ*ω*, where *ω* and Δ*ω* are the resonance frequency and the resonance width at half-max, respectively).

**Figure 3:**
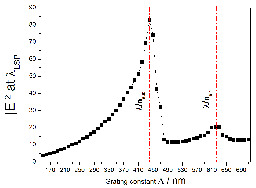
Calculated intensity of the local electric field at the maximum of the LSP mode wavelength versus the grating constant. Calculations are made by the FDTD method for gold discs (D = 100 nm and h = 50 nm).

Since the quality factor Q is increasing, the near-field intensity is expected to also increase. In the Figure 3, we present the FDTD calculation of the intensity of the local electric field (calculated at the maximum of the lattice mode wavelength) versus the grating constant. It can be seen that the maximum of intensity is obtained for Λ = 430 nm, corresponding to the calculated critical grating constant Λ_*c*_, where a strong long-range coupling occurs. A second maximum at Λ ∼ 620 nm, with a much lower intensity, is observed, and attributed to a grating order (0,±1) in air. The slight difference between the experimental and calculated Λ_*c*_ comes from the fact that in the calculations, the NSR is not taken into account [38,39].

We thus expect that the choice of the grating constant, in the context of SERS measurements, will impact significantly the Raman enhancement factor. However, in order to be able to compare the experimental SERS measurements versus the grating constant with the calculated REF, one has to take into account the calculated REF defined as REF_*calc*_ = | E(*ω_exc_*) | ^2^ * | E(*ω_RS_*) | ^2^ (*ω_exc_* and *ω_RS_* correspond to the laser excitation and Raman (Stokes side) angular frequency, respectively. We thus plotted the REF_*calc*_ versus the grating constant, by considering the two Raman lines at 1074 and 1585 cm^−1^, corresponding to characteristic Raman bands of 4-MBA molecules, for an incident wavelength at 633 nm ( excitation line used in our experiments).

**Figure 4:**
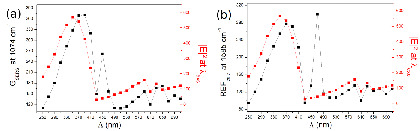
Calculated Raman enhancement factor (REF_*calc*_) versus the grating constant for the Raman line at 1074 cm^−1^ (**a**) and 1585 cm^−1^ (**b**). The REFs are superposed with | E(*ω_exc_*) | ^4^, calculated at 633 nm. The calculation are made for gold nanodiscs arrays with a diameter of D = 100 nm. The height of nanodiscs is h = 50 nm.

As displayed in Figure 4, the maximum of REF_*calc*_ is expected to be maximum at a smaller grating constant (Λ = 370 nm), compared to the maximum of intensity measured at *λ_LSP_* (at Λ = 430 nm). The REF_*calc*_ is also compared to | E(*ω_exc_*) | ^4^ at *λ_exc_* = 633 nm, which corresponds to an approximation often used for the estimation of the REF. It is seen that the maximum of | E(*ω_exc_*) | ^4^ (for an incident wavelength at 633 nm) is obtained for a smaller grating constant, compared to the maximum of REF_*calc*_. This difference was expected since one has to take into account the enhancement factor | E(*ω_RS_*) | ^2^ at *λ_RS_*, red-shifted compared to the enhancement factor | E(*ω_exc_*) | ^2^ at *λ_exc_*.

Finally, a “sharp” maximum is also observed at Λ = 450 nm for the 1074 cm^−1^ Raman line, and at Λ = 470 nm for the 1585 cm^−1^ Raman line. This difference in grating constant is due to the fact that the Raman emissions are located at different wavelengths. For instance, the Raman emission at 1585 cm^−1^ is more predominant for a higher grating constant since it corresponds to an LSP wavelength more red-shifted compared to those of a smaller Λ. These two additional maxima are attributed to the fact that the maximum of the LSP is precisely located at half way between the excitation line and the Raman lines (*λ_LSP_* = (*λ_exc_* + *λ_RS_*)/2), leading to an optimized Raman gain, as demonstrated in the reference [36].

**Figure 5:**
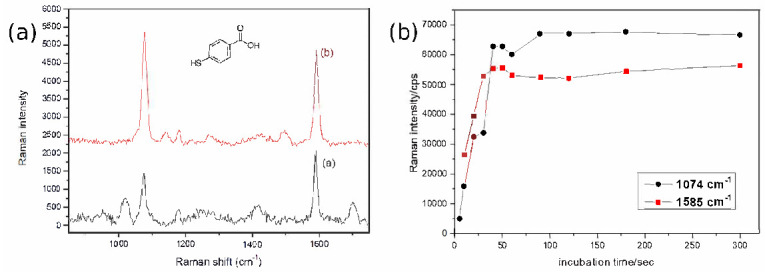
(**a**) Spontaneous Raman spectrum of 4-mercaptobenzoic acid (4-MBA) of (0.5 M) in DMSO solution (spectrum a), and surface enhanced Raman scattering (SERS) spectra of 4-MBA molecules adsorbed on a gold nanodisc array (concentration of 10^−4^ M, sepctrum b). Acquisition conditions for SERS: excitation wavelength *λ_exc_* = 632.8 nm, laser power P = 65 μW, acquisition time t = 30 s; (**b**) SERS intensity as a function of incubation time for the Raman bands at 1074 cm^−1^ and 1585 cm^−1^. Acquisition conditions: excitation wavelength *λ_exc_* = 632.8 nm, laser power P = 65 μW, acquisition time t = 30 s.

In the following, we investigate experimentally the impact of the grating constant on the Raman gain, by considering as the superstrate, the air. The spontaneous Raman spectrum was first characterized for a 0.5 M 4-MBA in a DMSO solution (Figure 5a). The Raman signature is mainly characterized by two intense Raman bands of 4-MBA located at 1074 and 1585 cm^−1^ (spectrum a), associated to CH out-phase bonding and to C=C symmetric stretching vibrations, respectively. The adsorption of the molecules onto monolayers is crucial in order to estimate experimentally the number of molecules contributing to SERS, and thus, the Raman enhancement factor (spectrum b, Figure 5a). In order to verify that 4-MBA molecules form monolayers onto the surface of gold nanoparticles, we recorded the SERS spectra of 4-MBA molecules on a gold nanodisc array (D = 95 nm, h = 50 nm, Λ = 320 nm), with different incubation times (molecular concentration of 10^−4^ M). Figure 5b shows the intensity of the Raman bands at 1074 cm^−1^ and 1585 cm^−1^ versus the incubation time adsorbed on a gold nanodiscs array. The SERS intensity increases and reaches its maximum after ∼50 s of incubation time. This intensity remains constant when the incubation time is increased. This result allows us to conclude that a monolayer of 4-MBA molecules is formed for an incubation time of 40–50 s. In the following SERS experiments, we will use an incubation time of 5 min in a solution of 10^−4^ M, in order to insure that the gold particles are fully covered by a monolayer of 4-MBA molecules.

Since the 4-MBA molecules form a monolayer at the particles surface, it is possible to estimate the order of magnitude of the number of adsorbed molecules (knowing the surface occupied by one molecule), and thus, the Raman enhancement factor (REF). The Raman enhancement factor per molecule is defined as [32,35]:

(2) $$REF=\frac{I_{SERS}/N_{SERS}}{I_{Ref}/N_{Ref}}$$

with N_*SERS*_ = (N × S_*NP*_)/S_*mol*_ and N_*Ref*_ = C × V_*eff*_ × N_*A*_.

In Equation (2), I_*SERS*_ corresponds to the integrated intensity of the Raman bands of the 4-MBA molecules, I_*Ref*_ the integrated Raman intensity corresponding to the spontaneous Raman spectrum recorded for a 0.5 M 4-MBA in a DMSO solution. N_*SERS*_ is the number of molecules occupied in the laser spot surface, N is the number of metallic nanoparticles under the laser spot area, S_*NP*_ is the surface occupied by one nanoparticle and S_*mol*_ is the surface occupied by one molecule of 4-MBA equal to 38.3 Å^2^. The laser spot surface has thus been estimated to 5 μm^2^ for a 100× microscope objective (N.A. 0.65). Therefore, we could estimate N_*SERS*_ for the different arrays investigated. N_*Ref*_ is the number of molecules excited in a volume V_*eff*_ of the laser waist for 0.5 M 4-MBA solution. N_*A*_ is the Avogadro number equal to 6.02 × 10^23^ mol^−1^. The volume of laser waist is estimated by considering a cone of apex angle defined by the numerical aperture of the microscope objective and the height of the focusing scope. Using a 100× microscope objective with a numerical aperture NA of 0.65, the volume of laser waist is assumed to be 5000 μm^3^, leading to a N_*Ref*_ value of ∼1.5 × 10^12^ molecules. This definition of the Raman enhancement instead of that given in Refs. [32,35] indeed overestimates slightly the gain by not accounting for the molecules on lateral part of the particles. However, we believe that it is more suitable because we are investigating self assembled molecules chemically adsorbed to the gold surface by the sulfur atom; few molecules are adsorbed on the side of the particles and play only a weak role. Indeed, computations show that the electromagnetic field should be weak on this part of the nanodiscs. Therefore, this definition does not change the conclusion concerning the evolution of the average enhancement with the grating constant which was the main goal of this paper.

**Figure 6:**
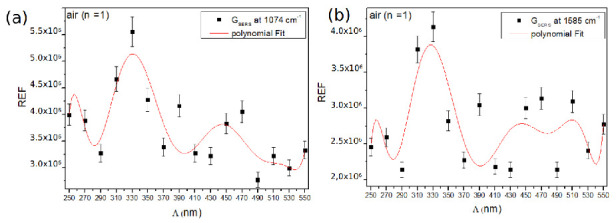
Experimental Raman enhancement factors REF_*exp*_ as a function of the grating constant for the Raman bands at 1074 cm^−1^ (**a**), and 1585 cm^−1^ (**b**). Acquisition conditions: excitation wavelength *λ_exc_* = 632.8 nm, laser power P = 65 μW, acquisition time t = 30 s.

The SERS signals were recorded at *λ_exc_* = 632.8 nm. Using a microscope objective with a 100× magnification, and a numerical aperture of N.A. = 0.9, the estimated zone of excitation was ∼5 μm^2^. The Figure 6 displays the Raman enhancement factor versus the grating constant for the Raman bands at 1074 and 1585 cm^−1^. The REF has been measured, on the order of 10^6^, in quite good quantitative agreements with recent works [40]. For both Raman bands, we observe that the maximum of REF is obtained for a grating constant of Λ = 330 nm, and not for Λ = 430 nm, for which it was observed a maximum of intensity at *λ_LSP_* (Figure 6a,b). The maximum of the experimental REF_*exp*_ at Λ = 330 nm is slightly different from the calculated REF_*calc*_ located at Λ = 370 nm (see the Figure 4). This discrepancy is attributed to the fact that the calculated values are extracted from the spectral profile of the near-field intensities, which reflect the calculated extinction spectra. The calculated spectra are slightly shifted compared to the experimental ones, and thus explain why the calculated REF_*calc*_ is maximum for a slightly higher grating constant. However, the experimental REFs_*exp*_ are also observed for smaller grating constants than Λ*_C_*, as confirmed by the FDTD calculations (compare Figure 6a,b and Figure 4).

It is noteworthy that the maximum of REF_*exp*_, obtained for a grating constant at Λ = 330 nm, is not considerably improved, compared to the REF_*exp*_ measured for other grating constants (Figure 6a,b). Indeed, a factor of two is observed compared to the lowest values of the experimental REF (for instance at Λ = 290 or 490 nm). However, a factor of the same order of magnitude (∼3) is also deduced from the calculations, between the maximum REF_*calc*_ (at Λ = 370 nm) and the minimum REF_*calc*_ (at Λ = 490 nm). Finally, if a clear maximum of REF_*exp*_ is observed for Λ = 330 nm, significant fluctuations of the REFs versus the grating constant are observed. The calculated REF_*calc*_ also displays some fluctuations, although less obvious (Figure 4). This is attributed to the fact that the enhancement factors are not optimized in air.

In order to improve the REF, one has to consider a symmetric environment. In other words, the refractive index of the substrate needs to be as close as possible to the refractive index of the superstrate. Indeed, in an asymmetric medium, the radiative patterns by the nanodiscs are mostly scattered inside the substrate. Therefore, the overlap between the grazing diffracted orders and the particle plasmon is limited. In a symmetric environment, the radiative pattern is expected to be symmetric, and thus with a higher coupling, resulting in a strongest near-field intensity [23]. In the following, we thus investigate the impact of the dielectric environment on the far-field and near-field optical response, as well as on the Raman enhancement factors in the context of SERS measurements.

**Figure 7:**
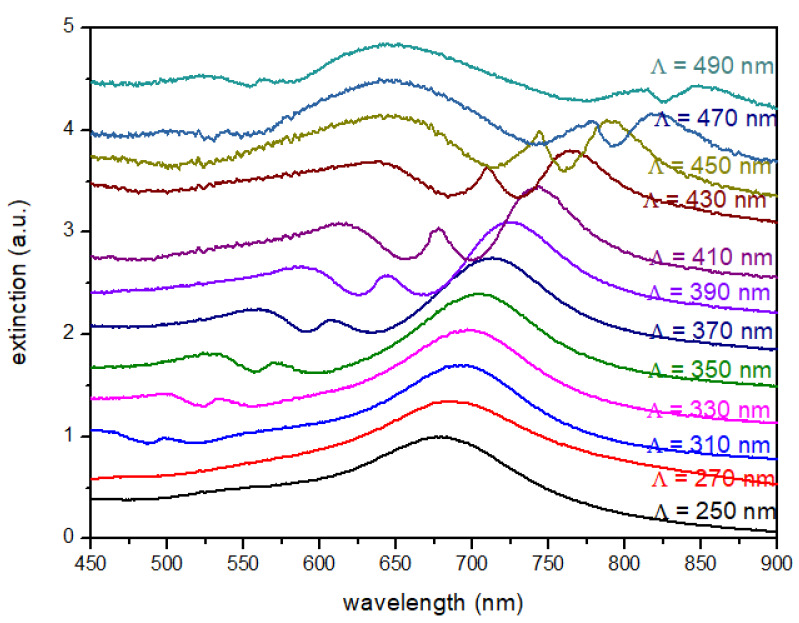
Extinction spectra recorded in oil (in transmission and normal incidence) for square arrays of gold nanodiscs with a diameter D = 100 nm. The grating constant varies from Λ = 250 to 490 nm. The height of nanodiscs is fixed to h = 50 nm.

**Figure 8:**
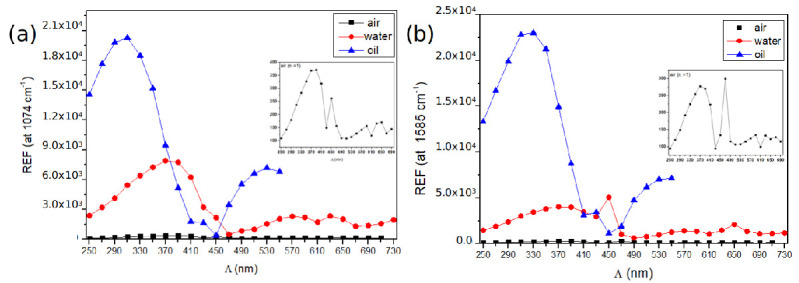
Calculated REFs_*calc*_ in air (n_*a*_ = 1), water (n_*w*_ = 1.33) and oil (n_*o*_ = 1.518) at: 1074 cm^−1^ (**a**) and 1585 cm^−1^ (**b**) for arrays of gold nanodiscs. Diameter of the disc is D = 100 nm, the height h = 50 nm and grating constant varying from Λ = 250 to 730 nm. The REFs are calculated using the FDTD method.

As the superstrate, we considered oil since its refractive index is very close from the substrate (n = 1.51). The Figure 7 displays the extinction spectra of the nanodiscs arrays, recorded in oil, with grating constants varying from Λ = 250 to 490 nm. As expected, a red-shift of the LSP resonances is observed, compared to the ones in air, due to a higher refractive index. The REF_*calc*_ has been calculated by taking into account the product of the square modulus of the electric field at *λ_exc_* = 632.8 nm and the square modulus of the electric field at 1074 cm^−1^ and 1585 cm^−1^ corresponding to $\lambda_{RS,1074}$ = 679 nm and $\lambda_{RS,1585}$ = 704 nm, respectively. Figure 8 displays the calculated REFs_*calc*_ for the Raman line at 1074 cm^−1^ (Figure 8a) and at 1585 cm^−1^ (Figure 8b). The REFs are also compared to the REFs calculated in air and water (as an intermediate dielectric medium, with a refractive index of n = 1.33).

For both Raman lines, the calculated REFs at maximum are at least 50 times higher in oil compared to air, and 4 times higher, compared to water. Moreover, the maximum of REFs_*calc*_ in oil corresponds to smaller grating constants (around Λ = 310 nm), compared to the calculated ones in air (around Λ = 370 nm). This can be explained by the fact that the LSP resonances in oil are more red-shifted compared to the excitation line, especially for higher grating constants. Thus, the maximum of REFs_*calc*_ is expected to be obtained for arrays with smaller grating constants, where the lattice mode wavelength is close to the excitation line at 633 nm. One can note that the maximum of REFs_*calc*_ is located at Λ = 310 nm for the 1074 cm^−1^ Raman line, and at Λ = 330 nm for the 1585 cm^−1^. This is attributed to the fact that the 1585 cm^−1^ Raman line corresponds to a higher wavelength compared to that of 1074 cm^−1^ Raman line. Finally, a second maximum of REF, with a lower value at Λ = 530 nm, is observed. This can be attributed to the diffracted (±1,±1) order in the substrate plane.

**Figure 9:**
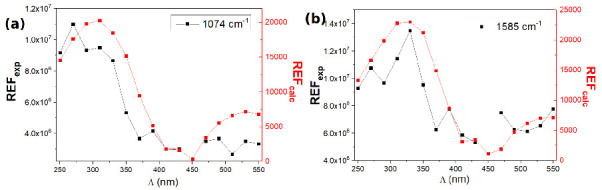
Experimental and calculated REF in oil for the Raman bands at 1074 cm^−1^ (**a**) and 1585 cm^−1^ (**b**).

The experimental SERS measurements in oil have been investigated using a microscope objective (50x, N.A. 0.9), with an excitation line *λ_exc_* = 632.8 nm and a laser power of 65 μW. The experimental REFs vary from 2 × 10^6^ (for Λ = 510 nm) to 10^7^ (for Λ = 270 nm). Figure 9 compares the experimental REFs versus the grating constant with the calculated REFs in oil. For both Raman lines, there is a good qualitative agreement between the experimental and calculated profile of the REFs versus the grating constant. In particular, the maximum of REF is obtained for a smaller grating constant for the 1074 cm^−1^ Raman line (at Λ = 300 nm), compared to the REF for the 1585 cm^−1^ Raman line (at Λ = 330 nm).

Note that the REFs_*exp*_ and REFs*_calc_* values are not quantitatively comparable. Although a chemical contribution on the Raman gain may contribute, it could not explain a difference of two orders of magnitude between the experimental and calculated REFs. Such discrepancy has been also pointed out by M. Banaee et al. [40], using the same molecular probe. They noted similar differences between simulation and experimental REF values, attributed to fabrication imperfections. This discrepancy could be explained by the fact that the simulations do not take into account any roughness of the discs surface. Indeed, this roughness is due to the thermal evaporation process before the lift-off step of the EBL fabrication of the samples. Therefore, it has been shown that a significant difference in REF can be observed between smooth (annealed) and roughened (non-annealed) samples. It has been shown that the calculated REF with roughened samples can be ∼100 times higher than that for a smooth sample. However, in our experiments, by considering the adequate grating constant in a symmetric environment, we show that the measured REF can be increased by one order of magnitude in comparison to those measured in air, and reaches values of the order of 10^7^, which represents high values to detect any molecular probes at very low concentrations.

## 3. Materials and Methods

Electron beam lithography: The substrates were using an electron beam lithographic system using a ZEISS scanning electronic microscope (SEM) [41]. A 100 nm thick layer of poly-methyl methacrylate electron resist was spin coated on glass substrates with an 80 nm layer of indium tin oxide (ITO). The desired structures were exposed to an electron beam. Chemical development, thermal vacuum coating with gold and a lift-off procedure followed, which led to regular arrays of gold nanodiscs of the desired geometrical parameters, 50 ± 5 nm height and 100 μm diameter on top of the ITO covered glass-substrate. This method allows us to control precisely the nanoparticle size, shape and inter-particle distance between nanoparticles. We have thus the ability to tune the plasmon resonance at any desired wavelength [41,42]. We choose isotropic gold discs in order to avoid any contribution of depolarization effect in Raman measurements, for instance affected by the anisotropy of gold nanorods.

UV–visible absorption spectroscopy: The plasmon bands were characterized by extinction micro-spectroscopy in the range of 400–900 nm. The spectrometer (LOT ORIEL model MS 260i) was coupled to an optical microscope (OLYMPUS BX 51) equipped with a 50× objective (numerical aperture N.A. 0.55).

Raman spectroscopy: The Raman experiments were made using a Jobin–Yvon LABRAM HR 800 Raman spectrometer. The source is an He-Ne laser (632.8 nm), focused on the sample, through a microscope equipped with a 100× objective (Olympus, N.A. 0.8). 4-mercaptobenzoic acid (4-MBA) molecules were used for all the SERS experiments. This molecule is characterized by self-assembled monolayers, when adsorbed on the surface of metallic nanoparticles. Indeed, the adsorption of these molecules takes place via the thiol group on the gold surface.

Finite Difference Time Domain (FDTD) calculations: Finite Difference Time Domain (FDTD) simulations were achieved using a developed 3D-code for the optical proprieties investigation. The code takes into account the periodicity of the structure in x and y directions via Bloch’s boundary conditions [43] and the upper and lower semi-infinite media in z direction through perfectly matched layer (PML) conditions of Berenger [44]. The implemented Critical Points Drude model [45] deals with the dispersive nature of gold and ITO using different fitted parameters to match experimental values. The structure is illuminated, with a plane wave, at normal incidence from the substrate. In the near-field, the normalized electric field intensity is calculated in the vicinity of the metallic nanoparticles, while the detector is placed far away from them for far-field simulations of extinction spectra.

## 4. Conclusions

It has been shown that a long-range coupling within a gold nanodisc array affects significantly the SERS intensity of a molecular probe (4-mercaptobenzoic acid). This type of coupling corresponds to the emergence of a new radiative order in the substrate plane. This interaction is maximum for a critical grating constant Λ_*c*_, when the plasmon mode wavelength is close to the Rayleigh anomaly position. As a consequence, the REF is strongly dependent on the grating constant. The maximum of REF is not obtained for a grating constant corresponding to a maximum of local electric field intensity, but systematically obtained for smaller grating constants regardless the environment. Experimental and theoretical values of the REF display that one needs to consider a symmetric environment, in order to optimize the REF. More importantly, it is demonstrated that the Raman gain of molecular probes can be improved with one order of magnitude in a symmetric medium (in oil) compared to SERS experiments in air, by considering the appropriate grating constant.
